# Applicability of on-site disinfection of personal protective equipment by ozone gas

**DOI:** 10.1016/j.heliyon.2023.e13360

**Published:** 2023-02-03

**Authors:** Shunji Ishiwata, Taishi Hibino, Tomoe Sakashita, Manami Nishioka, Tomomi Inoue, Takeshi Kotake

**Affiliations:** a Division of Medical Pharmaceutics and Therapeutics, Faculty of Pharmacy, Kindai University, Osaka, Japan; b Department of Pharmacy, Sakai City Medical Center, Osaka, Japan

**Keywords:** Ozone gas, Personal protective equipment, On-site disinfection, Pandemic, PPE, personal protective equipment, COVID-19, corona virus disease 2019, SARS-CoV-2, severe acute respiratory syndrome corona virus 2, CT, concentration-time, SCD, soybean-casein digest

## Abstract

On-site disinfection techniques are beneficial during a pandemic when there is a marked shortage of personal protective equipment (PPE), as experienced during the coronavirus disease 2019 outbreak. Ozone gas has been considered an alternative on-site disinfectant during a pandemic because it has antimicrobial activities, can be produced from air by electricity without the need for storage, and can be easily deactivated after use. However, ozone gas might become distributed at the lower layer because it has a larger molecular weight than air.

This study aimed to reveal the applicability of ozone gas for the on-site disinfection of PPE. The lockers meant for changing dresses were used as ozone gas exposure boxes, and the distribution of ozone was assayed. Considering that the determined ozone levels were not consistent in the types of ozone analysers, we studied the chemical and biological activities of ozone, which were evenly detected in the locker. The gown in the locker was also uniformly exposed to ozone. Results showed that ozone gas could be used for the on-site disinfection of PPE in a closed box, such as a locker. This finding is valuable during a pandemic when PPE is in short supply.

## 1 Introduction

Pandemics regularly occur throughout history, and the coronavirus disease 2019 (COVID-19) pandemic is the most recent one to affect mankind [1]. There are several types of pathogenic organisms, such as avian influenza virus, in addition to the coronavirus, which can cause an outbreak of a pandemic. These organisms have great diversity in their origin and sensitivity against disinfectants, thus making the preparation for and control of pandemics difficult. Given what the world has recently experienced during the COVID-19 outbreak, the supply of medical equipment, especially personal protective equipment (PPE), decreases during a pandemic because worldwide production and transportation are affected [2]. In these cases, it is recommended to disinfect and reuse medical equipment on-site, such as in hospitals [3].

The use of ozone gas was considered an alternative for on-site disinfection during the COVID-19 pandemic [4]. It has antiviral and antibacterial activities against a variety of pathogenic organisms. Furthermore, it can be easily produced from air by electricity without the need for storage and can be deactivated after use. Recently, ozone gas has been reported to decrease the infectivity levels of severe acute respiratory syndrome coronavirus 2 (SARS-CoV-2) [5]. We have previously developed and reported a visible ozone indicator that can be adjusted for disinfections against a range of pathogenic organisms, including SARS-CoV-2 [6]. The indicator can visualise the end point of the ozone exposure for the disinfection of each pathogenic organism. Dennis et al. [7] reported the durability of disposable N95 masks against ozone gas. The reactivity of ozone gas is so elevated that it has been assumed that ozone gas damaged not only the microorganisms but the structure or function of the exposed equipment until recently. Therefore, these reports increased the potential of ozone gas as a valuable on-site disinfectant; however, its distribution in closed air, which is important for uniform disinfection, remains obscure. The remaining concern is that ozone gas might distribute at the lower layer because of its larger molecular weight compared with air.

This study aimed to evaluate the applicability of ozone gas for on-site disinfection, such as in hospitals, in preparation for a pandemic. We studied the concentration and activity of ozone gas for on-site disinfection in lockers meant for changing dresses under various conditions.

## 2 Methods

**Fig. 1:**
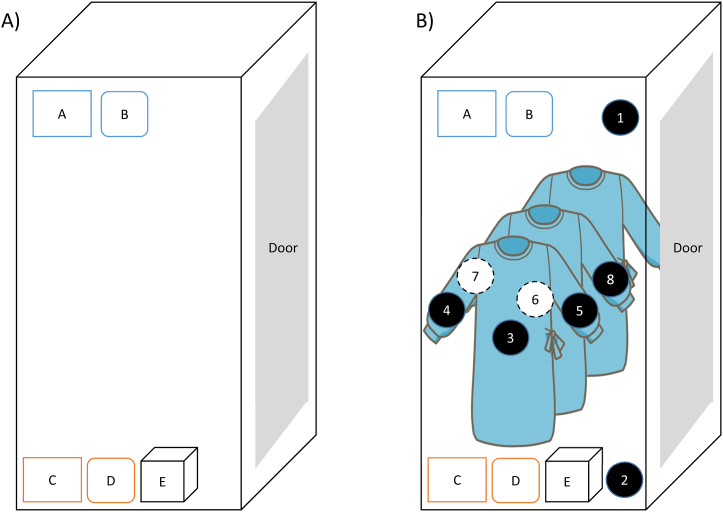
Ozone exposure locker. A) Illustration of the ozone exposure locker. Apparatus: A, thermohygrometer (upper); B, ozone meter (upper); C, thermohygrometer (lower); D, ozone meter (lower); E, ozone generator. B) Illustration of the ozone exposure of the gown in the locker. Apparatus: A, thermohygrometer (upper); B, ozone meter (upper); C, thermohygrometer (lower); D, ozone meter (lower); E, ozone generator. Ozone indicator: 1, upper air; 2, lower air; 3, abdominal of outer gown; 4, right sleeve of outer gown; 5, left sleeve of outer gown; 6, abdominal of centre gown; 7, right sleeve of centre gown; 8, left sleeve of centre gown.

The applicability of ozone gas for the on-site disinfection of PPE was evaluated in lockers meant for changing dresses. An ozone generator (Tamura Teco, Osaka, Japan) was placed at the bottom of a locker (460 mm × 520 mm × 1790 mm, Itoki, Tokyo, Japan) made of painted steel (Fig. 1A). The exposure level of ozone gas is expressed as a concentration–time (CT) value, which is obtained by multiplying the concentration (ppm) by the duration of exposure (minute) of ozone gas. We have previously shown that the chemical activity of ozone gas is dependent on the CT value irrespective of the concentration [6]. Ozone gas was generated under 20 °C and 50%–60% relative humidity in the closed locker. The ozone levels were determined by ozone analysers UV-100 (Eco Sensors, Saint Fe, NM, USA), which measured the absorbance at 254 nm, and TOM-10GII (Tamura Teco, Osaka, Japan), which used an electrochemical sensor. These ozone analysers were placed at the bottom of (lower area) and 1600 mm above the ozone generator (upper area) in the locker. The temperature and humidity in the locker were monitored by thermohygrometers (SwitchBot, Bao'an, P.R. China).

**Fig. 2:**
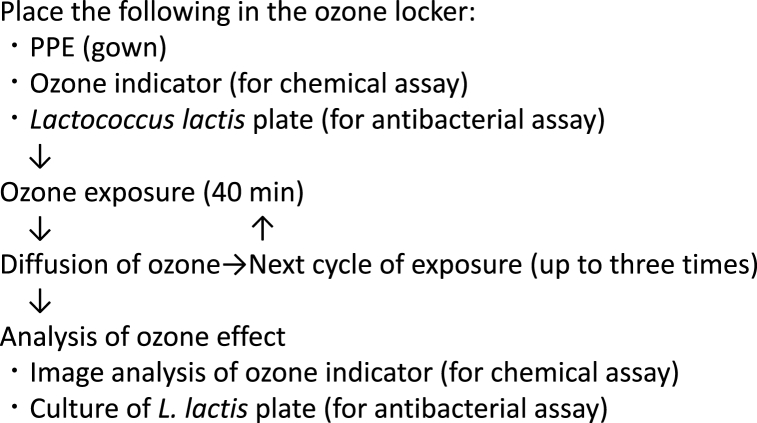
The procedure of experimental on-site disinfection of PPE by ozone gas.

The chemical activity of ozone gas was monitored by an ozone indicator that was previously prepared [6]. The intensity of the indicator decreased as the exposure level of ozone gas increased and was analyzed by image analysis software Image J (National Institute of Health, Bethesda, MA, USA). The activity of ozone gas was evaluated as the residual ratio, which was calculated as the ratio between the postexperiment densities of the colour and the preexperiment densities of the colour. Therefore, the elevated activity of ozone gas is measured as the decreased level of the residual ratio of the indicator. The indicators were set at the same level as the ozone analysers described above. The antibacterial activity of ozone gas was also evaluated using *Lactococcus lactis* (ATCC49032) as a model organism. The bacteria were cultured in soybean–casein digest (SCD) liquid media at 37 °C overnight, followed by a 10^5^-fold dilution and seeding on an SCD plate. The plate was placed at the same level as the ozone analysers and exposed to ozone as described above for 0, 8, 10, or 12 min. After culturing the bacteria overnight, the number of colonies was counted (Fig. 2).

The disposable gowns were purchased from Hakuzo (Osaka, Japan). Three gowns were hung on hangers placed in the locker (Fig. 1B). The ozone indicators were clipped to the gown of the abdominal portion and the cuffs of both sleeves of the gowns by a stapler. The indicators were stapled on the centre and exterior gowns. A 40-min exposure to ozone was performed and allowed to diffuse into air in a vacant room, and ozone production was reinitiated for the next experiment after the level of ozone in the locker was decreased to the baseline. The variation of the levels of residual rates was shown as relative standard deviation (RSD).

## 3 Results

**Fig. 3:**
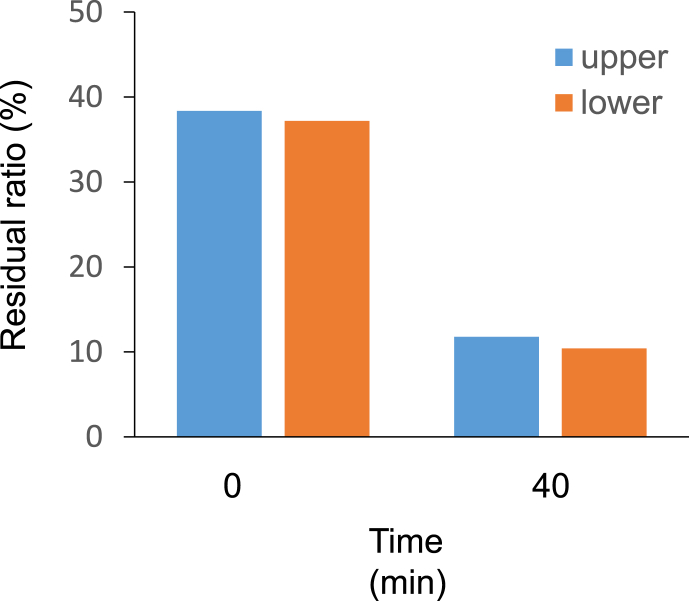
The chemical activity of ozone determined by the ozone indicator. The time indicates the exposure duration of ozone.

**Fig. 4:**
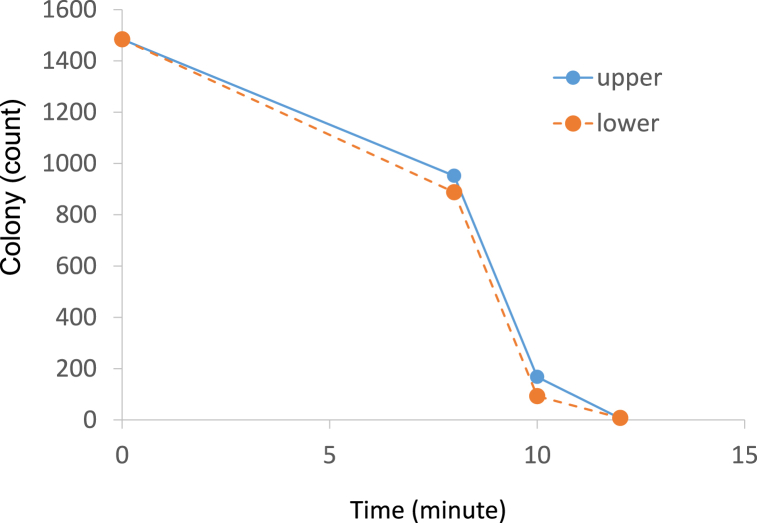
The biological activity of ozone determined by *Lactococcus lactis.* The time indicates the exposure duration of ozone before overnight incubation.

After the ozone generator, ozone analysers, thermohygrometers, and ozone indicator were placed in the locker, ozone production was initiated. The levels of ozone gas in the upper and lower regions increased time dependently. The ozone levels determined by ozone analysers UV-100 were the same in both regions during the experiment. However, the levels determined by electrochemical sensor TOM-10GII differed for the upper and lower regions. The ozone levels at the bottom were higher than those measured in the upper region. The residual ratio of the indicator in the upper and lower air is shown in Fig. 3. The number of bacterial colonies in the upper and lower areas decreased as the exposure time increased (Fig. 4). No difference was observed between the levels in the upper and lower regions.

For the next experiment, the three gowns were placed in the locker and then exposed to ozone gas for three cycles. The residual ratio of the gown after the first cycle of exposure was 69.5–86.1, and the mean and RSD were 77.4 and 7.4%, respectively (Table 1). The mean values of the residual ratio after 2 and 3 cycles of exposure were 66.9 and 68.4, respectively. The RSDs after two and three cycles were 5.8% and 3.9%, respectively.

**Table 1:** Variation of the residual ratio of the indicator in the locker.

Number	Position	Residual ratio (%)		
		Number of cycles		
		1	2	3
1	upper air	77.2	71.2	66.1
2	lower air	86.1	73.7	71.5
3	abdominal of outer gown	72.6	65.8	69.5
4	right sleeve of outer gown	75.8	68.5	65.4
5	left sleeve of outer gown	69.5	65.0	69.2
6	abdominal of center gown	73.4	64.7	69.6
7	right sleeve of center gown	83.7	63.7	71.5
8	left sleeve of center gown	80.5	62.4	64.4
Mean		77.4	66.9	68.4
RSD (%)		7.4	5.8	3.9

## 4 Discussion

The ability of the ozone gas to mix with air appeared to be in favour of the uniform disinfection of PPE. The uneven distribution of disinfectants leads to uneven disinfection. We have previously reported that an elevated level of an anticancer drug gas, namely, cyclophosphamide, was detected in the lower air compared with that in the upper air [8]. We showed here that the determined levels of ozone in the lower and higher regions were mixed value and dependent on the analysers. The two kinds of analysers were different in detecting mechanisms of ozone, which can be the reason for the difference. Therefore, we attempted to assay the activity instead of the ozone concentration. The chemical and biological activities of ozone gas were measured by the indicator and lactic acid bacteria, respectively. These activities were evenly detected and consistent with the ozone gas levels measured by the UV-100 ozone analyser.

The disposable gowns were placed in the locker to elucidate the applicability of ozone gas for the disinfection of PPE. The effective CT value for SARS-CoV-2 was reported to be 60 [5]. Therefore, the CT value of our experiment was set at approximately half of the effective value to estimate the activity of ozone. The RSDs of the residual ratio of the indicator in air and on the abdominal portion and both sleeves were less than 10%, thus suggesting that ozone reacted with the gowns evenly in the locker. Although the airway of ozone gas seemed to be narrowed by the gowns in the locker, it diffused uniformly.

The activity of ozone was lower during the first exposure cycle than during the second and third exposures. The gown used in this experiment was blue because of the use of phthalocyanine. Although phthalocyanine pigment is widely used in disposable equipment, it is believed to be sensitive to ozone gas [9]. Given that ozone gas can react with elements on the surface without penetration, it is reasonable to assume that it reacted with phthalocyanine on the surface during the first exposure, thereby reducing the level of the gas. Thereafter, the levels relatively increased because of the lack of pigment on the surface.

The on-site disinfection and reuse of PPE, such as gowns and disposable masks, was inevitable during the pandemic because of the insufficient supply of equipment even on ordinary days [2]. The European Centre for Disease Prevention and Control has recommended reusing PPE in the COVID-19 pandemic [10]. The practical guidelines for improvised disinfection using consumer-grade ozone generators and the durability of disposable N95 masks against ozone gas have been reported [7]. These data emphasise the possibility of on-site disinfection from pathogenic organisms.

Ozone gas has been regarded a potential disinfectant. However, there are concerns that ozone gas can be distributed at the lower layer in closed air because the molecular weight of ozone is approximately one-and-a-half times as large as that of air. Although the uniform distribution of ozone gas is essential for disinfection, there have been few reports on the distribution of ozone gas in closed air. We revealed that the chemical and biological activities of ozone gas is uniformly detected in closed air, thus removing the remaining concern on the use of ozone gas for disinfection. To the best of our knowledge, our study reports the applicability of ozone gas for disinfecting PPE for the first time. However, this research has limitations. We used an ozone indicator and *L. lactis* to estimate the activity of ozone because it is difficult to evaluate this on-site disinfection system experimentally by using pathogenic organisms. Although the effect of ozone on pathogenic organisms was dependent on the CT value, which is determined by the indicator, there has been no report on the direct correlation of the effect of ozone on pathogenic organisms with the sensitivity of the ozone indicator. Furthermore, the antiviral and antibacterial spectra of ozone remains to be determined for proper disinfection. We hope that this report will contribute to data on PPE reuse and on-site disinfection for future pandemics.

## Author contribution statement

Takeshi Kotake, Ph.D: Conceived and designed the experiments; Wrote the paper.

Shunji Ishiwata, Ph D; Taishi Hibino: Conceived and designed the experiments; Performed the experiments; Analyzed and interpreted the data; Wrote the paper.

Tomoe Sakashita; Manami Nishioka: Performed the experiments; Analyzed and interpreted the data; Contributed reagents, materials, analysis tools or data.

Tomomi Inoue, Ph D: Analyzed and interpreted the data; Contributed reagents, materials, analysis tools or data; Wrote the paper.

## Funding statement

Professor Takeshi Kotake was supported by Kindai University [All-Kindai University support project against COVID-19].

Shunji Ishiwata was supported by Tamura Teco [Tamura Teco].

## Data availability statement

The authors do not have permission to share data.

## Declaration of interest’s statement

The authors declare the following conflict of interests: S.I. received research grants from Tamura Teco Co., Ltd, Osaka, Japan. All other authors have no conflict of interest to declare.
